# Trauma-specific Grey Matter Alterations in PTSD

**DOI:** 10.1038/srep33748

**Published:** 2016-09-21

**Authors:** Linghui Meng, Jing Jiang, Changfeng Jin, Jia Liu, Youjin Zhao, Weina Wang, Kaiming Li, Qiyong Gong

**Affiliations:** 1 Huaxi MR Research Center (HMRRC), Department of Radiology, West China Hospital of Sichuan University, Chengdu 610041, China; 2 Department of Radiology, The Third Hospital of Hebei Medical University, Shijiazhuang 050051, China; 3 Zigong Mental Health Center, Zigong 643000, China

## Abstract

Previous studies have demonstrated that patients with posttraumatic stress disorder (PTSD) caused by different types of trauma may show divergence in epidemiology, clinical manifestation and treatment outcome. However, it is still unclear whether this divergence has neuroanatomic correlates in PTSD brains. To elucidate the general and trauma-specific cortical morphometric alterations, we performed a meta-analysis of grey matter (GM) changes in PTSD (N = 246) with different traumas and trauma-exposed controls (TECs, N = 347) using anisotropic effect-size signed differential mapping and its subgroup analysis. Our results revealed general GM reduction (GMR) foci in the prefrontal-limbic-striatal system of PTSD brains when compared with those of TECs. Notably, the GMR patterns were trauma-specific. For PTSD by single-incident traumas, GMR foci were found in bilateral medial prefrontal cortex (mPFC), anterior cingulate cortex (ACC), insula, striatum, left hippocampus and amygdala; and for PTSD by prolonged traumas in the left insula, striatum, amygdala and middle temporal gyrus. Moreover, Clinician-Administered PTSD Scale scores were found to be negatively associated with the GM changes in bilateral ACC and mPFC. Our study indicates that the GMR patterns of PTSD are associated with specific traumas, suggesting a stratified diagnosis and treatment for PTSD patients.

Posttraumatic stress disorder (PTSD) is the only major mental disorder with a known cause, i.e., an event that threatens one’s physical integrity or that of others^1^. Examples of traumatic events are natural disasters, accidents, combats, childhood abuse, sexual abuse and indirect exposure by learning that a close relative or a friend was exposed to trauma^2^. These events can be broadly classified into natural vs. man-made, intentional vs. non-intentional, or single-incident vs. repetitive or prolonged^3^,^4^,^5^,^6^. It has been reported that 60% of men and 50% of women in their lifetime will experience one or other kinds of trauma and approximately 20% of those trauma-exposed individuals will develop PTSD^7^. However, the risk of development of PTSD after trauma is subject to the trauma type. For instance, sexual abuse causes a higher rate of PTSD than other trauma categories^8^. Clinical trials also suggest that different traumatic events may interact with individual factors, leading to different physical and behavioral outcomes as well as different prevalence of PTSD^3^,^8^,^9^,^10^,^11^. For instance, Husarewycz *et al.* reported that natural disaster/terrorism is associated with cardiovascular disease, gastrointestinal disease and arthritis while combat-related trauma is not positively associated with any physical condition^9^. Moreover, medication and social support have been suggested to differentiate among trauma types^4^,^6^. For PTSD patients by prolonged traumas, the treatment of dissociation and interpersonal problems may be the first priority, rather than focusing on the impact of specific past events and the processing of specific traumatic memories in general PTSD patients^4^. These accumulated pieces of evidence indicate that there might be different areas of brain alterations or even mechanisms underlying PTSD by different kinds of trauma.

Magnetic resonance imaging (MRI) has proven to be a useful tool for exploration of the neural mechanisms of PTSD. For instance, high-resolution structural MRI combined with voxel-based morphometry (VBM) provides opportunities to investigate subtle structural abnormities^12^,^13^,^14^. As a well-established neuroimaging tool, VBM investigates the anatomical focal differences between two groups of brains using voxel-wise statistical parametric mapping. It usually consists of several steps including brain segmentation, registration/normalization, smoothing, statistical inferences and multiple comparison correction^15^. The resultant grey matter density reflects the local GM volume at a given voxel, making VBM capable of assessing focal volumetric differences of GM in the whole brain. As such, VBM has been widely used in structural MRI studies of various neuropsychiatric disorders^16^,^17^. Among them, quite a few studies are about the investigation of structural alterations in PTSD brains. However, partially due to the diversity and complexity of PTSD, their results are not quite consistent. For example, when compared with trauma-exposed controls (TECs), PTSD patients by fire disasters showed GM reductions (GMRs) in the left hippocampus^18^, while GMRs were observed in the left middle temporal gyrus (MTG) of PTSD patients by combats^19^. The inconsistency raises natural questions such as: “what are the general neuroanatomic alterations of PSTD brains and what are the trauma-specific ones?”

A couple of meta-analysis studies have been performed to address these questions. Boccia *et al.* conducted a functional MRI meta-analysis to assess the role of the traumatic events in PTSD^11^. Their results demonstrated that specific networks of brain areas underpin PTSD after different traumatic events. Meng *et al.* also reported that different kinds of trauma may have acted as a potential moderator or a source of method variance that contributed to the heterogeneous findings of PTSD studies^20^. Besides, there are three previously published VBM meta-analyses of grey matter changes in PTSD^12^,^20^,^21^. However, none of the above studies focused on the possible different effects exerted on PTSD brains by different traumas, leaving the trauma-specific morphometric alterations of PTSD a still open question.

We therefore in this study performed a VBM meta-analysis on GM changes of different PTSD subtypes, i.e., single-incident vs. prolonged trauma types^4^,^5^,^6^. Different from the previous reports^11^,^12^,^20^,^21^, we aimed to elucidate the trauma-specific GM volumetric alterations and their association with the demographics and clinical characteristics of PTSD patients. In specific, we carefully examined published VBM studies comparing PTSD with TEC and included 16 studies with various kinds of trauma (Table 1). We then used a coordinate-based meta-analytic technique called anisotropic effect-size signed differential mapping (AES-SDM) to explore the consistent GM volumetric alterations in PTSD. Subsequently, subgroup analyses were performed to investigate the trauma-specific GM changes of two trauma types, i.e., single-incident and prolonged trauma. Finally, association between GM alterations and Clinician-Administered PTSD Scale (CAPS) scores were also investigated.

## Results

### Included studies and sample characteristics

**Figure 1:**
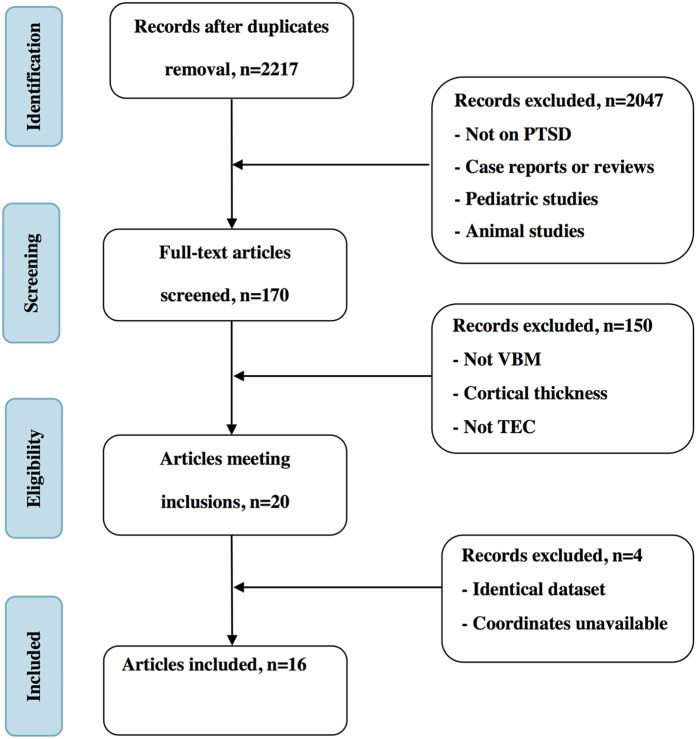
Flowchart of the literature selection in the present study.

Figure 1 shows the flowchart of our study selection procedure and the resultant studies after each step. As shown, our search strategy identified 2217 studies after removal of duplicates. No additional relevant articles were included by inspecting the references of included articles. Most studies (2047 out of 2217) did not meet the inclusion criteria based on inspections of their titles and/or abstracts. Two studies^22^,^23^ were excluded since they used the same dataset with another study^18^, whose statistical analysis, however, was stricter. A similar issue was found for studies by Chen and Zhang^13^,^24^, and the latter was also chosen due to stricter statistical analysis. After full-text inspection, 16 studies met our inclusion criteria and were included in our final meta-analysis^13^,^14^,^18^,^19^,^25^,^26^,^27^,^28^,^29^,^30^,^31^,^32^,^33^,^34^,^35^,^36^. These studies recruited 246 PTSD patients (mean age: 38.7 years old) and 347 TECs (mean age: 38.3 years old) in total. Their clinical and demographic information is summarized in Table 1. Notably, nine of these studies recruited patients by single-incident traumas including accident and natural disaster, and seven studies by prolonged traumas including combat, disease, rape, and refugee.

### Pooled meta-analysis

**Figure 2:**
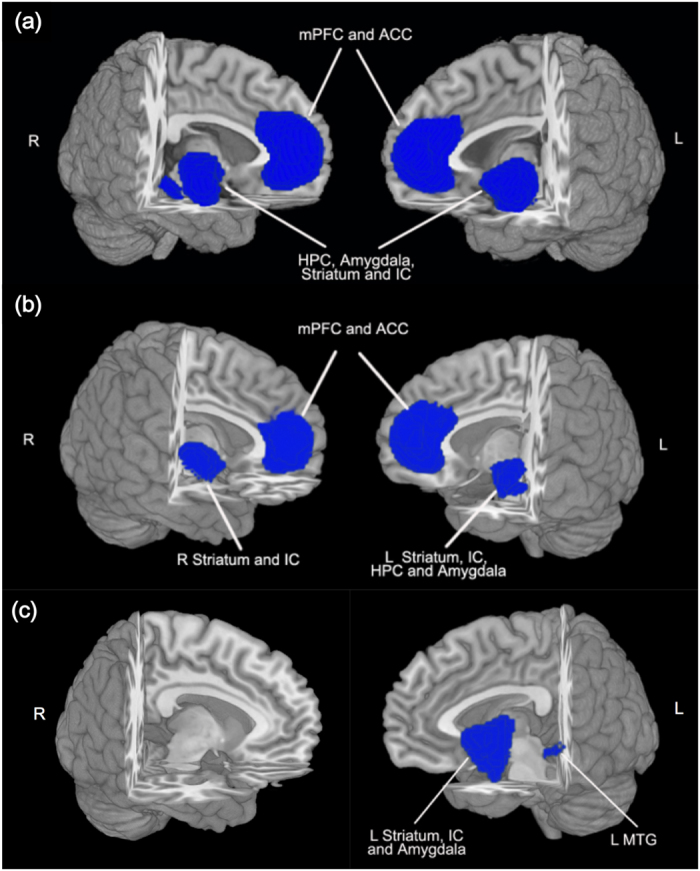
The brain regions exhibiting GM reduction in PTSD compared with TEC in the pooled meta-analysis (**a**), and subgroup analyses by single-incident (**b**) and prolonged trauma (**c**). The regions are displayed in a 3D brain, with part of the left or right hemisphere removed. Blue areas depict significant GMRs by AES-SDM in PTSD compared with TEC. Abbreviations: mPFC, medial prefrontal cortex; ACC, anterior cingulate cortex; HPC, hippocampus; IC, insula cortex; MTG, middle temporal gyrus; L, left; R, right.

As shown in Fig. 2a, a group comparison between PTSD and TEC revealed GMRs mostly in the prefrontal-limbic-striatal system, including bilateral anterior cingulate cortex (ACC), medial prefrontal cortex (mPFC), striatum, insula, amygdala, and hippocampus. Detailed information of general GMR regions (the MNI coordinates, SDM effect sizes, and Broadman areas) is summarized in Table 2. No GM increases were found in PTSD brains.

### Subgroup meta-analyses of different traumas

Subgroup analysis in PTSD patients by single-incident traumas (9 studies, 129 PTSD patients, and 137 TECs) revealed GMRs in bilateral mPFC, ACC, insula and striatum, left hippocampus and amygdala, as depicted in Fig. 2b. In contrast, subgroup analysis of PTSD patients by prolonged traumas (7 studies, 117 PTSD patients, and 210 TECs) revealed GMRs in the left insula, striatum, amygdala and MTG, (shown in Fig. 2c). For detailed information (e.g., the MNI coordinates, SDM effect sizes and Broadman areas), please refer to Table 2.

### Heterogeneity and publication bias analyses

Heterogeneity analysis showed that no regions in our pooled meta-analysis have significant heterogeneity between studies. Analysis of publication bias showed that the Egger test is insignificant for the right ACC (p = 0.264), the right mPFC (p = 0.409), the right striatum (p = 0.439), the right insula (p = 0.593), the right hippocampus (p = 0.957), the right amygdala (p = 0.524), the left mPFC (p = 0.083), the left striatum (p = 0.894), the left insula (p = 0.458), the left hippocampus (p = 0.457), the left amygdala (p = 0.626), except for the left ACC (p = 0.021).

### Sensitivity analysis

Whole-brain jack-knife sensitivity analysis showed that GMRs in bilateral ACC and mPFC were highly replicable and well preserved throughout all combinations. The findings in left striatum, insula, hippocampus, and amygdala remained significant in all but one combination, which is the same for the findings in right striatum, insula, and amygdala. The findings in the right hippocampus remained significant in all but two combinations.

### Meta-regression analysis

**Figure 3:**
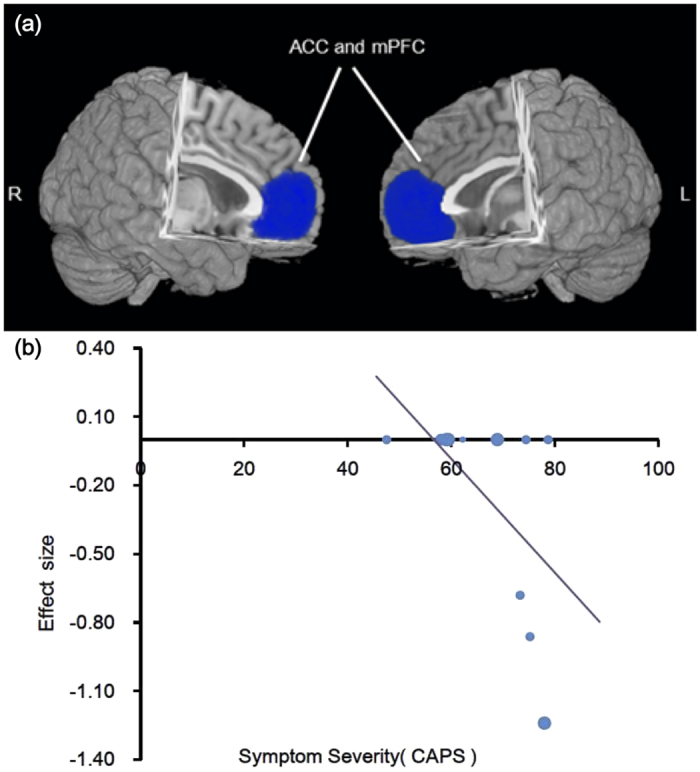
Association between CAPS and GM alterations in PTSD. (**a**) The brain areas associating with CAPS scores in PTSD patients; (**b**) relationship between CAPS scores and GM alterations of regions in a. The effect sizes were extracted from the peak of maximum slope significance. Each study is represented by a dot. The dot size reflects the sample size. Large dots are for studies with more than 20 patients; medium dots: 10–20 patients; and small dots: <10 patients. The blue areas depict significant GMRs by AES-SDM in PTSD compared with TEC. Abbreviations: mPFC, medial prefrontal cortex; ACC, anterior cingulate cortex; CAPS, Clinician-Administered PTSD Scale; L, left; R, right.

Symptom severity (CAPS scores) of PTSD patients was negatively associated with GM changes in bilateral ACC and mPFC, as shown in Fig. 3 (Z = −2.424, P < 0.0001). The percentage of female PTSD patients and the mean age of patients were not significantly associated with PTSD-related GM changes, at least not linearly.

## Discussion

In the present study, we conducted a voxel-wise meta-analysis using AES-SDM for general and trauma-specific GM alteration patterns of PTSD brains compared with those of TECs. Our pooled meta-analysis obtained a general pattern of GMR in PTSD compared with TEC, mostly in bilateral mPFC, ACC, striatum, insula, hippocampus, and amygdala. In terms of trauma-specific GM alteration patterns, PTSD patients by single-incident traumas were characterized by GMRs in bilateral mPFC, ACC, striatum, and insula, the left hippocampus, and amygdala. While patients by prolonged traumas demonstrated quite a different pattern of GMR, with most affected regions found in the left striatum, insula, amygdala and MTG. In addition, GM alterations in the bilateral ACC and mPFC were negatively correlated with the severity of PTSD symptoms as measured by CAPS.

### General GMRs in PTSD

Our pooled meta-analysis indicates that general brain regions affected by PTSD are mostly located in the prefrontal-limbic-striatal system. The prefrontal-limbic circuit is associated with fear conditioning and has been widely reported to exhibit both anatomical and functional deficits in PTSD patients^12^,^37^. These deficits may lead to inability to effectively control attention and respond to trauma-related stimuli. Accompanied with the deficits in top-down inhibitory control of PTSD patients, increased amygdala response promote trauma recollections and hyperarousal, and abnormal hippocampal function in learning and memory^38^. Meanwhile, striatal regions, playing critical role in behavior reinforcement and punishment, have also been widely reported in both structural and functional PTSD studies^39^,^40^. For example, a study of high-risk population of PTSD showed significantly GM volume alterations in the prefrontal-limbic-striatal circuit^41^. Consistent with the structural MRI changes, functional MRI studies in survivors who had recently experienced severe emotional trauma also demonstrated functional alterations in the prefrontal-limbic and striatal areas, and attenuated connectivity among limbic and striatal networks^42^,^43^. Our analysis replicates the structural findings and further supports that, deficits in the prefrontal-limbic circuit are the core neural correlates of PTSD.

### Specific GMRs by single-incident traumas

Single-incident traumas are acute stressful events, limited in time^5^. For PTSD caused by this type of trauma, our subgroup meta-analysis revealed that GMRs were primarily located in the bilateral mPFC, ACC, striatum, insula, the left hippocampus and amygdala. Both human and animal studies have demonstrated that even mild traumatic stress can rapidly impair the function of mPFC and improve the function of amygdala and hippocampus^12^,^44^. When the stress goes to severer, hippocampal functions can also be impaired^45^, accompanied with hyperfunction of amygdala and striatum^47^. In a word, severe acute stressors may impair PFC-mediated cognitive/emotional functions and switch the control of behaviour and emotion to more primitive brain circuits, i.e., the limbic system and striatum^44^. For ACC, a vital brain region associated with fear conditioning, its functional disruption may facilitate the core symptoms of PTSD. As shown in a longitudinal study^48^, PTSD patients with aggravated symptoms showed accelerated atrophy in the ACC, which is consistent with our finding that symptom severity (CAPS scores) of PTSD patients was negatively associated with GM changes in bilateral ACC and mPFC. Taken together, mPFC-limbic-striatal system may be the primary affected brain system associated with acute single-incident traumas.

### Specific GMRs by prolonged traumas

In contrast to the above subgroup, PTSD patients having prolonged traumas revealed a different pattern of GMR, which mainly involved left striatum, insula, amygdala and MTG, but not the mPFC structures. This difference may be due to that the long-term fear expression induced by prolonged and repetitive trauma exposure may not be mediated by the mPFC-limbic network^49^, but the striatum and insula instead^39^. Increased functional connectivity between striatum and insular cortices during repeated exposure to the traumatic memories has been reported^39^. We believe that these hyper activities are closely related to the GMRs in the striatum and insula, although it is unclear whether the GMRs induced the hyper functional activity or the opposite. In addition, PTSD patients with prolonged traumatic experiences often show a dissociation syndrome, in contrast to PTSD patients by acute or single traumatic events^37^. Interestingly, this dissociation syndrome is reported to involve striatum, insula and amygdala^50^,^51^, which happen to exhibit GMR in our analysis. Finally, the long-term repeated exposure to different kinds of trauma may impair memory processing in the patients, as shown by the GMR in MTG. All these results indicate that PTSD by prolonged traumas exhibits a very different GMR pattern compared with single-incident induced ones, suggesting different neural mechanism may underlie PTSD by prolonged traumas.

### Differences between subtypes

Our results demonstrated that for PTSD patients by acute single-incident traumas, GMRs generally appear in the mPFC-limbic-striatal system while for PTSD by prolonged traumas, GMRs are dominant in the limbic-striatal structures. This difference indicates that there may be different brain mechanisms underlying PTSD by different traumas. One hypothesis is that for PTSD by acute single-incident traumas, mPFC is easily impaired due to its susceptibility to uncontrollable stress^12^,^44^. Accompanied are hyper-functions of primitive brain structure on emotional regulation, i.e., amygdala, insula and striatum^44^. These primitive regions, however, can also be damaged after longtime exposure to stress, as evidenced by our findings of the GMRs in PTSD by prolonged traumas and some other studies^39^,^52^.

Notably, there are some potential confounds that might contribute to the resultant differences in GMRs of the subtypes, such as gender and illness duration. Males with PTSD have been found to exhibit increased activation in left ACC during extinction recall compared with female PTSD patients^53^. Besides, some studies on GM changes in PTSD have provided evidence that illness duration was significantly associated with right hippocampal volume^54^.

### Limitations and future directions

Our results revealed that the regions of GMR in PTSD are subjected to trauma. However, the findings should be interpreted in light of several possible limitations of the present study. First, only sixteen studies are included in this study. This limited number of studies prevents us from more detailed subgroup meta-analyses. It may also affect the generalisation of our results. Second, it should be considered that gender and illness duration differences might confound the between-trauma findings in the PTSD group. Third, whether the GMRs are predispositions or consequences of PTSD is difficult to elucidate. Further studies with longitudinal structural changes may be necessary to disentangle this. Fourth, some original studies included in our meta-analysis did not exclude PTSD with other psychiatric disorders (e.g., major depressive disorder). Finally, publication bias was shown in the left ACC, and relevant findings should be interpreted with caution.

## Conclusion

In this study, we performed a quantitative voxel-wise meta-analysis of GM changes in PTSD by different traumas using AES-SDM, and found that GMR regions were generally located in the prefrontal-limbic-striatal system. Notably, subgroup analyses revealed that the GMR patterns were associated with specific trauma categories. This study provides further evidences of different neural correlates underlying PTSD by different traumas, and suggests that stratified diagnosis and treatment of PTSD are necessary in clinics.

## Methods

### Study inclusion and exclusion

A systematic search strategy^12^ was used to identify relevant studies indexed by PubMed, Cochrane Library, EBSCO, Web of Science, and ScienceDirect by November 2015. Keywords were set as (1): “posttraumatic stress disorder” or “PTSD” or “stress” or “trauma” or “maltreatment” or “assault” or “war” or “combat” or “accident” or “disaster” or “veteran” or “abuse”, crossed with (2): “voxel-based morphometry” or “VBM” or “morphometry” or “volumetric” or “grey matter”. References in resultant PTSD review and meta-analysis articles were manually examined for possible inclusion.

Studies were included according to the following criteria: (1) used VBM to analyze brain GM changes in PTSD patients; (2) compared PTSD patients with TECs; (3) clearly reported traumatic types. For studies reported both corrected and uncorrected results, only the corrected ones were used in the subsequent analyses.

A candidate study was excluded if (1) it belonged to reviews, case reports or meta-analysis studies; (2) full-text record is non-English or unavailable; (3) recruited patients were younger than 18 years old; (4) the included patients had co-morbidity such as headache, traumatic brain injury or other physical diseases; (5) results were based on small volume correction; (6) different thresholds were used for different brain regions, and (7) peak coordinates of reported brain regions or the trauma type could not be determined.

### Quality assessment

The quality of an included study was independently assessed by two authors (L., Meng and J., Jiang), using a checklist (See Supplementary Table S1) adapted from previous meta-analytic studies^55^,^56^. This 12-item checklist consists of quality assessments for diagnostic procedures, demographic characterization, sample size, imaging and analysis technique, and consistency between conclusions and results. Each item was scored 1, 0.5 or 0 if the corresponding criterion was fully met, partially met or unfulfilled, respectively. Finally, consensus scores were obtained and are summarized in Table 1.

### Meta-analysis of regional differences in GM

Regional differences in GM between PTSD patients and TECs were analyzed using AES-SDM (http://www.sdmproject.com). This is a well-established meta- analysis toolkit with following features: (1) reconstruction of positive and negative differences in the same signed differential map to avoid any voxel appearing significant in opposite directions; (2) using effect sizes to combine reported peak coordinates with statistical parametric maps; (3) applying complementary analyses such as sensitivity, subgroup and meta-regression analysis to assess the robustness and heterogeneity of the results^57^. In addition, anisotropic kernels were adopted to assign different values to the different neighboring voxels based on the spatial correlation between them, allowing exhaustive and accurate meta-analysis^58^.

A pooled meta-analysis using all included studies was performed first for the general GM alteration of PTSD by various traumas. Then, two subgroup meta-analyses by different trauma types (i.e., single-incident and prolonged trauma) were conducted for the trauma-specific GM alteration. For all meta-analyses, the statistical significance of each voxel was determined using standard randomization tests^57^.

### Heterogeneity and publication bias analysis

In the pooled meta-analysis, the statistical heterogeneity of individual clusters between studies was examined using a random effect model with Q statistic^57^. We examined the possibility of publication bias for GM changes using Egger test^59^.

### Sensitivity analysis

In order to test the replicability of the results, a systematic whole-brain voxel-wise jack-knife sensitivity analysis was performed^57^. Pooled analysis repeated the main statistical analysis for sixteen times, discarding one different study each time. If a brain region remained significant in all or most of the combinations of studies, it was considered as highly replicable^60^.

### Meta-regression analysis

Several relevant socio-demographic and clinical characteristics were listed in Table 1, including the percentage of female PTSD patients in each study, the mean age of patients and the CAPS scores. Their potential effects towards the GM alterations were explored using meta-regression^57^. Notably, the time since trauma could not be explored since only a few studies (less than nine) reported such information^60^. In order to minimize the detection of spurious regions, we reduced the p-value to 0.0005 for abnormality detection in both the slope and one of the extremes of the regressor and discarded findings not from the main analysis^60^.

## Additional Information

**How to cite this article**: Meng, L. *et al.* Trauma-specific Grey Matter Alterations in PTSD. *Sci. Rep.* **6**, 33748; doi: 10.1038/srep33748 (2016).

## Supplementary Material

Supplementary Information

## Figures and Tables

**Table 1:** Demographic and clinical characteristics of subjects in the included 16 studies.

Study	Subjects (females, n)		Age (years)		Time since trauma	Severity (scale type)	Comorbidity of other psychiatric disorder	Quality scores (out of 12)	Drug status	Trauma
	PTSD	TEC	PTSD	TEC						
Bryant^25^	13(NA)	13(NA)	NA	NA	75.7 m	75.2 (CAPS)	5 patients with depression 1 patient with panic disorder	9	n = 5 SSRIs	Accident
Chao^26^	21(0)	20(0)	35.9	35.2	NA	59.1 (CAPS)	18 patients with major depression	11	n = 6 serotonergic antidepressants	Combat
Cortese^27^	20(1)	25(1)	30.5	30.6	NA	59.4 (CAPS)	6 patients with depression 3 patients with panic disorder 1 patient with generalized anxiety	11	Drug naive	Combat
Eckart^28^	20(0)	19(0)	36.2	34.1	>10y	68.9 (CAPS)	15 patients with major depression	11.5	n = 1 antidepressants	Refugee
Felmingham^29^	21(NA)	17(NA)	NA	NA	66 m	78 (CAPS)	11 patients with depression 1 patient with panic disorder	9.5	n = 5 SSRIs	Accident
Hakamata^30^	14(14)	100(100)	45.6	47.1	266d*	NA	Negative	10.5	No psychotropic medication during last month	Cancer-related disease
Herringa^19^	13(2)	15(1)	28.9	30.1	NA	47.5 (CAPS)	1 patient with generalized anxiety	10	Drug naive	Combat
Kasai^14^	18(0)	23(0)	52.8	51.8	NA	73.3 (CAPS)	NA	10.5	NA	Combat
Li^18^	12(8)	12(8)	34.56	33.25	6–8 m	43.12 (DEQ)	2 patients with major depression	11	Drug naive	Fire disaster
Nardo^31^	15(3)	17(6)	43.33	41.59	3 m–6y	14.60 (DES)	Negative	11	n = 1 tricyclic antidepressants	Accident
Nardo^32^	21(6)	22(6)	41.7	40.8	2.5y	67.9 (TAQ)	Negative	11	NA	Accident
Rocha-Rego^33^	16(9)	16(9)	43.3	44.9	3y	NA	16 patients with major depression	10	n = 16 antidepressants	Accident
Sui^36^	11(11)	8(8)	25.55	27.50	45 m	74.45 (CAPS)	Negative	10	Drug naive	Rape
Tan^34^	12(0)	14(0)	37.6	40.9	2y	58.1 (CAPS)	Negative	10	No psychotropic medication during last two years	Mine disaster
Yamasue^35^	9(4)	16(6)	44.6	44.4	5–6y	62.2 (CAPS)	1 patient with major depression 2 patients with panic disorder	10	No psychotropic medication during last two years	Accident
Zhang^13^	10(0)	10(0)	40.8	34.3	187–190 d	78.72 (CAPS)	Negative	10	Drug naive	Mine disaster

Abbreviations: PTSD, Posttraumatic stress disorder; TEC, Trauma-exposed control without PTSD; CAPS, Clinician-Administered PTSD Scale; DEQ, Distressing Event Questionnaire; DES, Dissociative Experience Scale; TAQ, Trauma Antecedent Questionnaire; NA, not available; SSRIs, selective serotonin reuptake inhibitor medications.

^*^Time since the breast cancer surgery.

**Table 2:** Regional GM differences between PTSD patients and TEC subjects.

Brain regions (PTSD < TEC)	MNI coordinates			SDM z score	P value	Voxels, n	Cluster breakdown (voxels, n)
	X	Y	Z				
Pooled meta-analysis of all included studies							
L anterior cingulate/paracingulate gyri, BA32	−2	46	12	−2.494	0.000001311	3495	L anterior cingulate/paracingulate gyri, BA10,11, 24, 25, 32(867)
							L median cingulate/paracingulate gyri, BA24,32(135)
							L superior frontal gyrus, medial, BA9, 10, 11, 24, 32(676)
							L superior frontal gyrus, medial orbital, BA10, 11(304)
							L gyrus rectus, BA11(13)
							R anterior cingulate/paracingulate gyri, BA10,11, 25, 32(747)
							R superior frontal gyrus, medial, BA9,10 (369)
							R superior frontal gyrus, medial orbital, BA10, 11(325)
							R median cingulate/paracingulate gyri, BA32(53)
							R gyrus retcus, BA11(6)
L lenticular nucleus, putamen, BA48	−28	−2	−2	−2.427	0.000002563	1701	L lenticular nucleus, putamen, BA48 (849)
							L insula, BA48 (282)
							L amygdala, BA20, 28, 34, 36, 48 (227)
							L hippocampus, BA20, 28, 34, 35(94)
							L superior temporal gyrus, BA34, 48 (152)
							L olfactory, BA34, 48 (87)
							L parahippocampus, BA34,36(10)
R insula, BA48	40	−2	−4	−1.805	0.000422657	1040	R insula, BA48(513)
							R lenticular nucleus, putamen, BA48 (251)
							R superior temporal gyrus, BA20, 21 (209)
							R amygdala, BA34, 36, 48 (67)
R hippocampus, BA20	36	−30	−10	−1.515	0.002690315	68	R hippocampus, BA20 (61)
							R parahippocampus, BA20,37 (7)
Subgroup meta-analysis of single-incident trauma							
L superior frontal gyrus, medial. BA32	−8	52	18	−2.338	0.000006437	2902	L superior frontal gyrus, medial, BA9, 10, 11,32(830)
							L anterior cingulate/paracingulate gyri, BA10, 11, 24, 25, 32(756)
							L superior frontal gyrus, medial orbital, BA10 (119)
							L cingulum(92)
							R superior frontal gyrus, medial, BA9,10, 32 (436)
							R anterior cingulate/paracingulate gyri, BA11, 24, 25, 32(505)
							R superior frontal gyrus, medial orbital, BA11 (126)
							R cingulum(38)
R insula, BA48	40	−12	8	−1.487	0.001923442	518	R insula, BA48(200)
							R lenticular nucleus, putamen, BA48 (267)
							R striatum(51)
L insula, BA48	−38	−10	−2	−1.614	0.00083065	388	L insula, BA48(133)
							L lenticular nucleus, putamen, BA48 (105)
							L striatum, (93)
							L hippocampus, BA20 (38)
							L amygdala, BA34 (19)
Subgroup meta-analysis of prolonged trauma							
L lenticular nucleus, putamen, BA48	−32	0	−2	−1.921	0.000012875	1314	L lenticular nucleus, putamen, BA11, 34, 48 (459)
							L insula, BA38, 47, 48 (470)
							L striatum, (204)
							L amygdala, BA20, 34, 36, 38, 48 (114)
							L olfactory cortex, BA34, 48(48)
							L temporal pole, superior temporal gyrus, BA34, 38(19)
L middle temporal gyrus, BA21	−62	−32	−4	−1.186	0.003495157	26	L middle temporal gyrus, BA21(26)

Abbreviations: BA, Brodmann area; PTSD, posttraumatic stress disorder; TEC, trauma-exposed control; L, left; R, right; SDM, signed differential mapping.
