# Supplementary material for: Trauma-specific Grey Matter Alterations in PTSD
Source: Sci Rep. 2016 Sep 21;6:33748. doi: 10.1038/srep33748 (PMC5030628; doi:10.1038/srep33748)
Supplement: Supplementary Information [file srep33748-s1.pdf]

Trauma-specific Grey Matter Alterations in PTSD

Linghui Meng, Jing Jiang, Changfeng Jin, Jia Liu, Youjin Zhao, Weina Wang, Kaiming Li and Qiyong Gong

Table S1. Imaging Methodology Quality Assessment Checklist

Score (0/0.5/1)

Category 1: subjects

1. Patients were evaluated prospectively, specific diagnostic criteria were applied, and demographical data were reported;

2. Healthy comparison subjects were evaluated prospectively, psychiatric and medical illnesses were excluded, and demographical data were reported;

3. Important confounds (e.g., age, gender, intelligence quotient, handedness, socioeconomic status, medication status, comorbidity) were controlled either by stratification or statistically;

4. Sample size per group ≥20;

Category 2: methods for image acquisition and analysis

5. All neuroanatomic measurements were made blind to group assignment and to subjects' identity;

6. Measures for brain structures were reported;

7. MRI slice-thickness ≤3mm;

8. The imaging technique was clearly described so as to be reproducible;

9. The measurements were clearly described so as to be reproducible;

10. Coordinates reported in a standard space;

Category 3: results and conclusions

11. Statistical parameters for significant and important non-significant differences were provided;

12. Conclusions were consistent with results and limitations are discussed.

TOTAL /12
